# An unusual case of silent acute ST-elevation myocardial infarction following amphetamine use

**DOI:** 10.12669/pjms.294.3500

**Published:** 2013

**Authors:** Julia Chia-Yu Chang, Chian-Ze Peng, Chorng-Kuang How, Mu-Shun Huang

**Affiliations:** 1 Julia Chia-Yu Chang, MD, Emergency Department, Taipei Veterans General Hospital, National Yang-Ming University, School of Medicine, Taipei, Taiwan.; 2 Chian-Ze Peng, MD, Emergency Department, Taipei Veterans General Hospital, National Yang-Ming University, School of Medicine, Taipei, Taiwan.; 3 Chorng-Kuang How, MD, Emergency Department, Taipei Veterans General Hospital, National Yang-Ming University, School of Medicine, Taipei, Taiwan.; 4 Mu-Shun Huang, MD, Emergency Department, Taipei Veterans General Hospital, National Yang-Ming University, School of Medicine, Taipei, Taiwan.

**Keywords:** Amphetamine, Chest pain, Silent myocardial infarction

## Abstract

We report a case of silent acute ST-elevation myocardial infarction associated with amphetamine use in a 62 years old diabetic man. The patient was devoid of chest pain and had a normal cardiac enzyme analysis at the initial presentation. A routine electrocardiogram demonstrated acute inferior wall ST-elevation myocardial infarction. Coronary angiography confirmed a total occlusion of the posterior lateral branch of right coronary artery. The patient underwent successful percutaneous transluminal coronary angioplasty with stent placement. Amphetamine abuse may play a role in acute myocardial infarction. Adverse cardiovascular manifestations of amphetamine can occur with sudden overt chest pain or present insidiously. In view of the potential association of amphetamine and myocardial infarction, physicians should not rely only upon clinical symptoms. This report highlights the diabetic patients with amphetamine abuse should undergo a routine electrocardiogram in such circumstances.

## INTRODUCTION

Risk of acute coronary syndrome in patients with amphetamine intoxication has been described. Amphetamine abuse may play a role in acute myocardial infarction. Chest pain is major symptom of potential amphetamine-associated adverse cardiovascular effects. However, adverse cardiovascular manifestations of amphetamine can occur with sudden overt clinical symptoms or present insidiously. Here, we report a case of silent acute ST-elevation myocardial infarction associated with amphetamine use in a 62-year-old diabetic man.

## CASE REPORT

A 62 years old man was brought by ambulance to our emergency department (ED) with the presentation of transient change in consciousness after amphetamine smoke inhalation. He had a history of diabetes mellitus and recreational drug use. At arrival, he was alert with no diaphoresis and denied having chest pain or chest tightness. Vital signs included a blood pressure of 105/73 mmHg, a pulse rate of 114 beats per minute, a respiratory rate of 22 breaths per minutes, and a core body temperature of 36.4C. Physical examination revealed bilateral pupil size of 2.5 mm with intact light reflex and unremarkable on cardiovascular, respiratory, and abdominal system.

**Fig.1:**
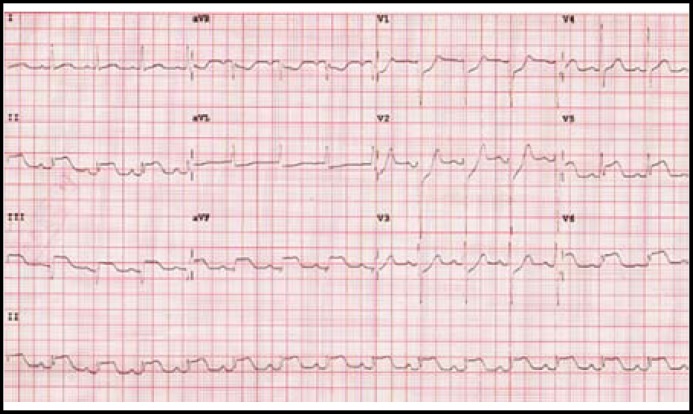
A 12-lead electrocardiogram on admission revealed ST-segment elevation in leads II, III, aVF, V_5_ and V_6_ with reciprocal ST-segment depression in V_1-3_

**Fig.2:**
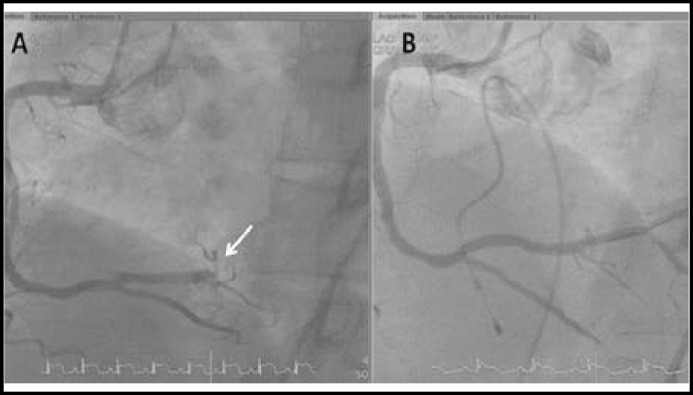
The left anterior oblique view of conventional coronary angiography delineated the RCA in the pre- and postangioplasty stages. A, A total occlusion of the RCA-PL (arrow). B, RCA-PL in postangioplasty stage showing normal contrast perfusion

The laboratory reported white cell count 11,700/mm^3^ and blood glucose level 290 mg/dL. Cardiac enzyme analysis showed a creatine kinase (CK) of 139 U/L (reference range, 0-140 U/L), CK-MB of 15U/L (reference range; <5 U/L), and troponin I of <0.04 ng/mL (reference range; 0-0.05 ng/mL). Surprisingly, a routine 12-lead electrocardiogram (ECG) demonstrated ST-segment elevation in leads II, III, aVF, V_5_ and V_6_ with reciprocal ST-segment depression in V_1-3_ (Fig.1). Primary coronary angiography was performed immediately and revealed single vessel disease with a total occlusion of the posterior lateral branch of right coronary artery (RCA-PL). The patient underwent successful percutaneous transluminal coronary angioplasty with stent placement (Fig.2).

Serial cardiac enzyme analysis confirmed a myocardial infarction with a peaking CK value of 2241 U/L (9.4%MB form) at 12 hours after ED arrival. The urine levels of amphetamine and methamphetamine collected at the time of presentation were 437 and >5,000 ng/mL, respectively, detected by gas chromatography-mass spectrometry. Screening of urine for cocaine metabolites, benzodiazepine, barbiturates, opiates, phencyclidine, cannabis, and 3,4-methylenedioxy-*N*-methylamphetamine were negative. The rest of the hospitalization course was uneventful.

## DISCUSSION

Amphetamine is a synthetic central nervous system stimulant which releases and blocks the reuptake of catecholamine resulting in a hyperadrenergic state.^1^ Risk of acute coronary syndrome in patients with amphetamine intoxication has been described. An acute coronary syndrome was diagnosed in 25% of patients presenting to the ED with chest pain after methamphetamine use.^2^ Amphetamine abuse may play a role in acute myocardial infarction. A population based epidemiologic study of hospitalised young adults indicates a modest, but significant, association between amphetamine abuse and acute myocardial infarction.^3^ The population attributable risk suggests that amphetamine abuse is responsible for 0.2% of acute myocardial infarction.^3^

However, the occurrence of ST elevation myocardial infarction after amphetamine use has been reported rarely in the literature.^1^^,^^2^^,^^4^ The possible explanations for amphetamine-induced myocardial ischemia include coronary vasospasm, catecholamine-mediated platelet aggregation, increase in shear stress with subsequent rupture of asymptomatic atherosclerotic plaques, and increased myocardial oxygen demand.^1^^,^^2^^,^^4^^,^^5^ Our patient had significant coronary artery stenosis on coronary angiogram, suggesting rupture of atherosclerotic plaque and thrombosis may have accounted for amphetamine-induced myocardial infarction.

Chest pain is major symptom of potential amphetamine-associated adverse cardiovascular effects. The rate of cardiac-related chest pain is 17% in patients with chest pain associated with methamphetamine and cocaine use in a chest pain observation unit.^6^ However, adverse cardiovascular manifestations of amphetamine can occur with sudden overt clinical symptoms or present insidiously.^4^ Our patient had no symptoms suggesting an acute coronary insult. It has been noted that autonomic neuropathy in diabetes mellitus leads to disturbed cardiac perception and thus may play a role in silent myocardial infarction.^7^

In view of the potential association of amphetamine and myocardial infarction, physicians should not rely only upon clinical symptoms. This report highlights the diabetic patients with amphetamine abuse should undergo a routine ECG in such circumstances.
